# Germline variant testing in serrated polyposis syndrome

**DOI:** 10.1111/jgh.15791

**Published:** 2022-02-18

**Authors:** Aisling Murphy, Joyce Solomons, Peter Risby, Jessica Gabriel, Tina Bedenham, Michael Johnson, Nathan Atkinson, Adam A Bailey, Elizabeth Bird‐Lieberman, Simon J Leedham, James E East, Sujata Biswas

**Affiliations:** ^1^ Translational Gastroenterology Unit, Oxford NIHR Biomedical Research Centre University of Oxford Oxford UK; ^2^ Oxford Centre for Genomic Medicine, Nuffield Orthopaedic Centre Oxford University Hospitals NHS Foundation Trust Oxford UK; ^3^ Oxford Regional Genetics Laboratories, Churchill Hospital Oxford University Hospitals NHS Foundation Trust Oxford UK; ^4^ New Zealand Familial Gastrointestinal Cancer Registry Auckland City Hospital Auckland New Zealand; ^5^ Intestinal Stem Cell Biology Lab, Wellcome Centre for Human Genetics University of Oxford Oxford UK; ^6^ Gastroenterology Department Buckinghamshire Healthcare NHS Trust UK

**Keywords:** colon, genetics, polyposis

## Abstract

**Background and Aim:**

Serrated polyposis syndrome (SPS) is now known to be the commonest polyposis syndrome. Previous analyses for germline variants have shown no consistent positive findings. To exclude other polyposis syndromes, 2019 British Society of Gastroenterology (BSG) guidelines advise gene panel testing if the patient is under 50 years, there are multiple affected individuals within a family, or there is dysplasia within any of the polyps.

**Methods:**

A database of SPS patients was established at the Oxford University Hospitals NHS Foundation Trust. Patients were referred for genetic assessment based on personal and family history and patient preference. The majority were tested for a hereditary colorectal cancer panel including MUTYH, APC, PTEN, SMAD4, BMPR1A, STK11, NTLH1, POLD1, POLE, GREM1 (40‐kb duplication), PMS2, and Lynch syndrome mismatch repair genes.

**Results:**

One hundred and seventy‐three patients were diagnosed with SPS based on World Health Organization 2019 criteria between February 2010 and December 2020. The mean age of diagnosis was 54.2 ± 16.8 years. Seventy‐three patients underwent genetic testing and 15/73 (20.5%) were found to have germline variants, of which 7/73 (9.6%) had a pathogenic variant (MUTYH *n* = 2, SMAD4 *n* = 1, CHEK2 *n* = 2, POLD1 *n* = 1, and RNF43 *n* = 1). Only 60% (9/15) of these patients would have been recommended for gene panel testing according to current BSG guidelines.

**Conclusions:**

A total of 20.5% of SPS patients tested were affected by heterozygous germline variants, including previously unreported associations with CHEK2 and POLD1. This led to a change in management in seven patients (9.6%). Current recommendations may miss SPS associated with germline variants, which is more common than previously anticipated.

## Introduction

Serrated polyposis syndrome (SPS) is a clinical phenotype characterized by the presence of multiple serrated polyps. Serrated polyps are a heterogeneous group of lesions with a saw‐toothed histological appearance of the crypt epithelium and include sessile serrated lesions (SSLs), SSL with dysplasia, traditional serrated adenoma (TSA), unclassified serrated adenoma, and hyperplastic polyps.^1^ It is thought that serrated polyps account for 15–35% of colorectal cancers (CRCs) by means of the serrated pathway, which involves somatic BRAF or KRAS gene mutation and subsequent MLH1 promotor methylation leading to microsatellite instability.^2^, ^3^, ^4^ The World Health Organization (WHO) has recently updated the diagnostic criteria for SPS^5^ to include either of the following:

- ≥ 5 serrated polyps ≥ 5 mm in size proximal to the rectum with ≥ 2 being ≥ 10 mm; or
- > 20 serrated polyps of any size throughout the bowel with ≥ 5 proximal to the rectum.

Serrated polyposis syndrome is associated with a significantly higher lifetime risk of CRC,^6^, ^7^, ^8^ up to 7% at 5 years.^9^ The prevalence of SPS is reported to be between 0.06% and 0.4% in primary colonoscopy screening cohorts and between 0.31% and 0.8% in fecal occult blood/fecal immunochemical test programs.^10^, ^11^, ^12^, ^13^, ^14^, ^15^

The British Society of Gastroenterology (BSG) have recently updated their guidance for genetic testing in SPS and recommend testing in patients with any of the following:

- age < 50 years;
- multiple affected patients within a family; and
- dysplasia within any of the polyps.

There is an increased risk of CRC among first‐degree relatives of patients with SPS suggesting a genetic cause, but less than 3% of SPS cases can be explained by known germline variants.^16^, ^17^, ^18^ In a study of 20 SPS families, a germline variant in RNF43, an inhibitor of the Wnt pathway, was reported in two unrelated individuals with SPS.^19^ To date, pathogenic RNF43 variants have been reported in nine SPS patients from seven families.^19^, ^20^, ^21^, ^22^, ^23^ However, there is no strong link between RNF43 mutation and serrated polyposis, with a prevalence of only 1.5–2.5% among SPS patients.^16^ Moreover, the missense mutations identified have not been consistent across cases.

There may be an overlap between the SPS phenotype and other hereditary cancer syndromes for which the genetic cause is known. MUTYH‐associated polyposis is caused by a biallelic variant in the MUTYH gene. One study found that 3/17 (18%) of individuals with biallelic MUTYH variants fulfilled the criteria for SPS, but these patients also had multiple adenomas.^24^ Conversely, in a study of SPS patients, a biallelic MUTYH variant was identified in only 1/126 patients and this patient had over 40 adenomas.^25^ A link with Cowden syndrome (PTEN variant) has also been made.^26^, ^27^ In a study of 127 PTEN variant carriers, 39% (*n* = 27) of those who underwent colonoscopy had hyperplastic polyps and 23% (*n* = 16) met the criteria for SPS. In addition, multiple serrated polyps have been found in individuals with SMAD4,^28^ BMPR1A,^29^ and GREM1^30^ variants, which may represent an overlap between the SPS clinical phenotype, juvenile polyposis, and hereditary mixed polyposis syndrome.

Other possible genes with reported links to SPS include FBLN2,^31^ EPHB2,^32^ ATM, PIF1, TELO2, XAF1, and RBL1.^19^

Cohort studies in which gene panel testing was performed in SPS patients have shown a very low yield for significant variants. In a study of 64 SPS patients, germline testing for BMPR1A, SMAD4, PTEN, MUTYH, and GREM1 genes found one monoallelic MUTYH and one non‐functional PTEN variant, but no pathogenic variants.^33^ Similarly, 29 patients fulfilling WHO criteria 1 for SPS had genetic testing for MUTYH, APC, and PTEN and no germline variants were found.^34^ The low yield of pathogenic Mendelian variants intimates a polygenic or alternative pathogenesis for this common polyposis. This may account for why SPS was not included in the 2019/2020 National Genomic Test Directory, which specifies genomic tests commissioned by the NHS for rare and inherited diseases.^35^

The aim in this study was to determine the yield of genetic variants in the Oxford SPS cohort and to determine whether current BSG recommendations would have identified patients with a significant germline variant.

## Methods

A database of patients with SPS was established in February 2010 at the Oxford University Hospitals NHS Trust. It includes patients who were diagnosed or referred to the tertiary referral center between February 2010 and December 2020. A retrospective review was conducted of all patients fulfilling WHO 2019 criteria for SPS in this database. Patient demographics, colonoscopy findings, histopathology, personal and family history, smoking status, and outcomes of genetic testing were obtained from the database and review of medical records.

Patients were referred for genetic assessment based on personal and family history, and patient preference. All patients referred underwent genetic counseling and genetic risk assessment. Patients were consented using a standard genetic test consent form. Some patients were tested through the 100 000 Genome Project, which utilized a specific test consent form.

Panel testing was introduced in Oxford in 2014. This initially consisted of a 17‐CRC/polyposis gene panel: APC, BMPR1A, CDH1, CHEK2, KIT, MLH1, MSH2, MSH6, MUTYH, PALB2, PMS2, POLD1 (exons 8–13), POLE (exons 9–14), PTEN, SMAD4, and STK11. The gene panel used has been refined over time to include the genes with the most clinical utility. A 12‐gene panel (APC, BMPR1A, MLH1, MSH2, MSH6, MUTYH, NTLH1, POLD1 [exons 8–13], POLE [exons 9–14], PTEN, SMAD4, and STK11) was in use from 2016 to 2018, with the addition of GREM1 (40‐kb duplication) in 2017 and PMS2 in 2018, to form the current 14‐gene panel that is aligned with the UK Cancer Genetics Group (UK‐CGG) consensus guideline on CRC/polyposis testing.^36^

Hereditary Cancer Solutions from Sophia Genetics, a Data Driven Medicine Platform, has been used since January 2018 to produce a custom panel and process the data in order to identify variants in cancer‐susceptibility genes. Next‐generation sequencing (NGS) was performed with a sensitivity > 99% for bases covered to a minimum depth of 50×. Multiplex ligation‐dependent probe amplification (MLPA) analysis was also conducted on MLH1 and MSH2 genes. Before January 2018, Haloplex Target Enrichment System was used and NGS had a sensitivity of > 99% for bases covered to a minimum depth of 30×. Target regions covered by NGS to a depth < 30× were analyzed by Sanger sequencing in the following genes: APC, BMPR1A, MLH1, MSH2, MSH6, POLD1 (exons 8–13), POLE (exons 9–14), PTEN, and SMAD4. MLPA analysis was performed on APC, MLH1, MSH2, MSH6, and GREM1. Before the introduction of gene panels, specific genes and specific variants were tested depending on the patient's history and phenotype using sequencing and MLPA. Whole genome sequencing (WGS) was performed with analysis of an extended 29‐gene panel on some patients enrolled in the 100 000 Genomes Project.

## Results

One hundred and seventy‐three patients were diagnosed with SPS based on WHO 2019 criteria between February 2010 and December 2020. The clinical characteristics are outlined in Table 1. The mean age of diagnosis was 54.2 ± 16.8 years (range 18–82 years). A total of 50.9% (*n* = 88) were female. 98 individuals fulfilled WHO criterion I (56.6%), 25 (14.5%) fulfilled criterion II, and 50 (28.9%) fulfilled both criteria I and II. The median number of polyps found was 15 (median of 7 polyps in the right colon and 3 in the left colon). The median size of a polyp was 9 mm and 25.4% of patients had polyps containing dysplasia (44/173).

**Table 1:** Characteristics of patients with serrated polyposis syndrome

		WHO type 1	WHO type 2	WHO types 1 and 2	Total
		*n* = 98 (56.6%)	*n* = 25 (14.5%)	*n* = 50 (28.9%)	*n* = 173
Sex	Female	54 (55.1%)	9 (36%)	25 (50%)	88 (50.86%)
	Male	44 (44.9%)	16 (64%)	25 (50%)	85 (49.13%)
Age (years)	Mean ± SD	62.4 ± 15.8	61.9 ± 16.3	51.5 ± 17.7	59 ± 17
Age at diagnosis (years)	Mean ± SD	57.4 ± 15.4	56 ± 16.9	47 ± 17.4	54.2 ± 16.8
Dysplasia in serrated polyp	Yes	22 (22.45%)	5 (20%)	15 (30%)	44 (25.4%)
	No	76 (77.55%)	20 (80%)	35 (70%)	129 (74.6%)
Colorectal cancer history	Yes	18 (18.37%)	2 (8%)	9 (18%)	29 (16.76%)
	No	80 (81.63%)	23 (98%)	41 (82%)	144 (83.24%)
Smoking	Smoker	17 (17.35%)	8 (32%)	12 (24%)	37 (21.39%)
	Ex‐smoker	30 (30.61%)	7 (28%)	14 (28%)	51 (29.48%)
	Never smoker	21 (21.42%)	6 (24%)	11 (22%)	38 (21.97%)
	Unknown	30 (30.61%)	4 (16%)	13 (26%)	47 (27.17%)

WHO, World Health Organization.

A total of 16.73% (29/173) of patients were diagnosed with CRC. Of the patients with CRC, the majority (69%, *n* = 20) were diagnosed with CRC at the time of SPS diagnosis. Eight patients (27.6%) were diagnosed with CRC before their diagnosis of SPS. A total of 55.2% (16/29) were right‐sided cancers and 44.8% (13/29) were left‐sided. A total of 6.9% of CRC patients (2/29) had metastatic disease. One patient was found to have a second metachronous tumor at the time of their SPS diagnosis. Only two (1.2% of the cohort) were diagnosed with CRC during follow up after their SPS diagnosis. One of these patients was diagnosed with metachronous CRC 5 years after the initial diagnosis of CRC and SPS before polyp clearance, and the second patient was diagnosed with CRC 39 months after diagnosis of SPS. Patients had a mean number of 2.2 ± 1.5 surveillance colonoscopies (range 1–8). Median follow‐up time was 35 months (interquartile range 15–52.5) from diagnosis of SPS to the date of their most recent colonoscopy. A total of 21.4% (*n* = 37) were current smokers.

**Figure 1:**
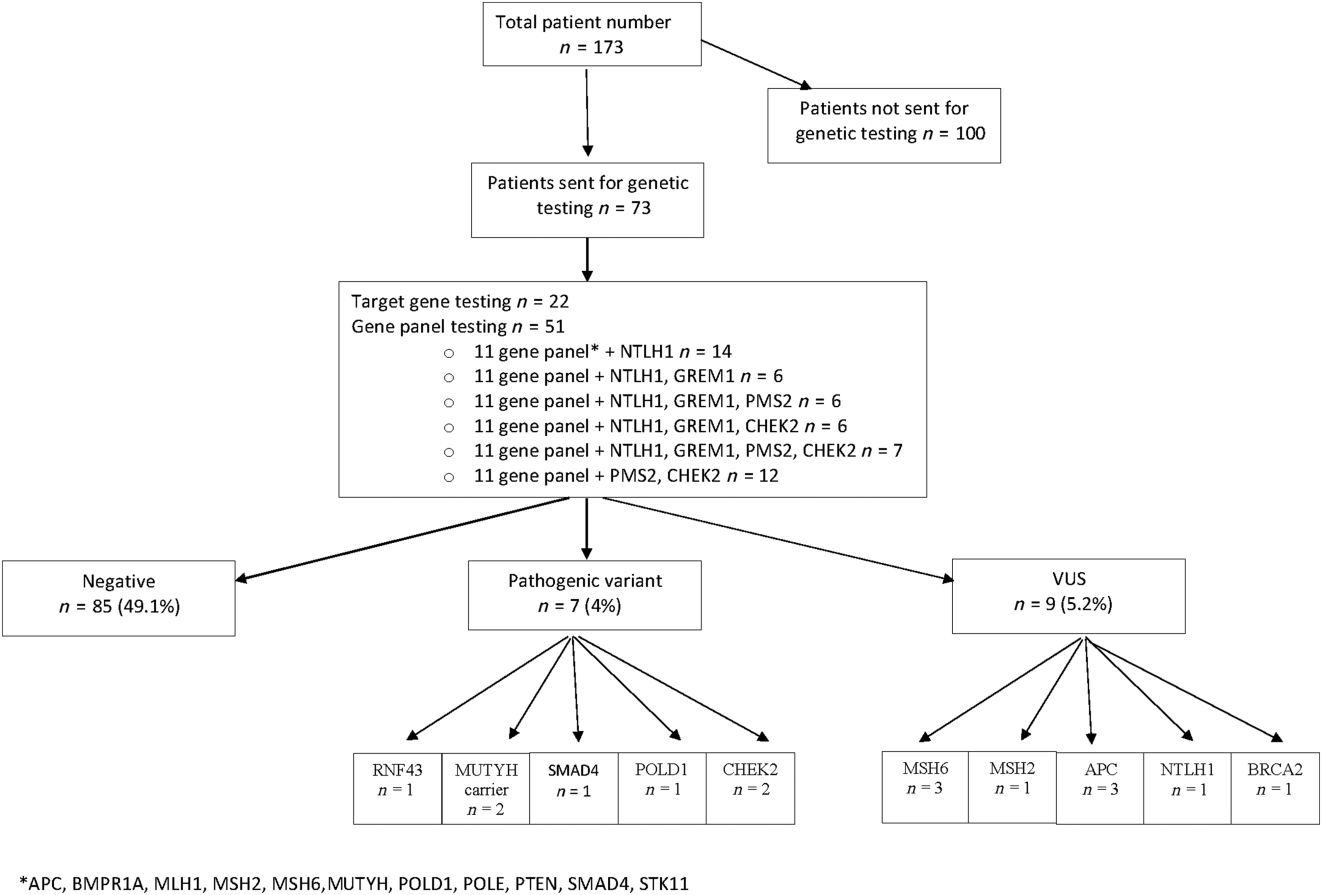
Results of genetic testing in serrated polyposis syndrome cohort. VUS, variants of unknown significance.

Out of 173 patients, 73 (42.2%) underwent genetic testing (Fig. 1). The majority (69.9%, *n* = 51) were tested using a CRC/polyposis gene panel. Twenty‐two patients (30.1%) underwent target gene testing. This includes the nine patients who underwent WGS with a 29‐gene panel as part of the 100 000 Genomes Project. Table 2 outlines the characteristics of patients who were genetically tested.

**Table 2:** Characteristics of serrated polyposis syndrome patients who underwent genetic testing

		Total
		*n* = 73
WHO type	Type 1	43 (58.9%)
	Type 2	10 (13.7%)
	Types 1 and 2	20 (27.4%)
Sex	Female	45 (61.6%)
	Male	28 (38.4%)
Age (years)	Mean ± SD	57.2 ± 16.6
Age at diagnosis (years)	Mean ± SD	52.1 ± 16.4
Dysplasia in SP	Yes	20 (27.4%)
	No	53 (72.6%)
Colorectal cancer history	Yes	11 (15.1%)
	No	62 (84.9%)
Smoking	Smoker	16 (21.9%)
	Ex‐smoker	15 (20.5%)
	Never smoker	20 (27.4%)
	Unknown	22 (30.1%)

WHO, World Health Organization.

Fifteen patients (8.6% of entire cohort, 20.5% of those who were tested) had a germline genetic variant and seven of these patients (4% of entire cohort, 9.6% of those tested) had a pathogenic variant in a gene known to be associated with cancer predisposition. Table 3 shows the differences in polyp characteristics in patients found to have a genetic variant. The genetic variants found are outlined in Table 4.

**Table 3:** Characteristics of serrated lesions in patients with and without a germline variant

	All SPS patients	Any germline variant	Pathogenic variant
Median no. of SP per patient	15	23	23
Median no. of SP in right colon	7	11	12
Median no. of SP in left colon	3	4	5
Median size of SP	9 mm	9 mm	9 mm
No. of patients with dysplasia	44/173 (25.4%)	8/15 (53%)	4/7 (57.1%)

SPS, serrated polyposis syndrome.

**Table 4:** Details of genetic variants identified and characteristics of patient and family

Gene affected	Pathogenic variant	WHO SPS type	Age at diagnosis	History of CRC (age)	Family history of CRC (age)	Personal cancer history	Family history of extraluminal cancer
RNF43	c.471 del G (exon 5) p.(Thr158fs) *Pathogenic variant*	I, II	68	No	Brother (30s) Mother (50s) Paternal grandmother (60s)	Prolactinoma	No
MUTYH	c.1187G > A	I, II	32	No	No	No	Prostate (mat grandfather) Lung (pat grandmother)
	p.(Gly396Asp)						
	*Pathogenic variant*						
APC	c.646‐4T > G						
	*Uncertain variant*						
MUTYH	c.1187G > A	I	70	No	No	No	No
	p.(Gly396Asp)						
	*Pathogenic variant*						
SMAD4	c.455‐2A > G	I	78	Yes (58)	No	No	Brain tumor (sister)
	*Pathogenic variant*						
POLD1	c.946G > A	1	70	No	Father (47)	Endometrial cancer (54)	Breast (sisters ×2, mat aunt, pat cousin)
	p.(Asp316Asn)						
	*Pathogenic variant*						
CHEK2	c.1427C > T	I	34	No	Great grandfather	No	Breast (mat grandmother)
	p.(Thr476Met)						
	*Pathogenic variant*						
CHEK2	c.1100delC	I	68	No	Father (64)	Breast cancer (64)	Breast (mother)
	p.(Thr367fs)						
	*Pathogenic variant*						
MSH6	c.1054G > A	II	30	No	Father (43)	No	Ovarian (pat grandmother)
	p.(Val352Ile)						
	*Uncertain variant*						
MSH6	c.2398G > C	I	59	No		No	No
	p.(Val800Leu)						
	*Uncertain variant*						
MSH6	c.3026A > T	II	36	No	Father (49) Paternal great aunt (80) Paternal great uncle (60)	Breast cancer (34)	Melanoma (pat aunt) Lung (pat grandfather)
	p.(Lys1009Ile)						
	*Uncertain variant*						
MSH2	c.835C > G,	I	37	Yes (28)	No	No	Breast (mother)
	p.(Leu279Val)						
	*Uncertain variant*						
APC	c.3479C > A	I, II	52	No	Paternal uncle (70s)	Prostate (57)	Lung (mat grandmother)
	p.(Thr1160Lys)						
	*Uncertain variant*						
APC	c.2486C > T	I, II	38	No	No	No	No
	p.(Thr829Ile)						
	*Uncertain variant*						
NTHL1	c.512C > T	I, II	32	No	Maternal aunt Maternal grandfather	No	Lung (mat uncle)
	p.(Thr171Met)						
	*Uncertain variant*						
BRCA2	c.7820C > T	I	60	Yes (58, 74)	Father (65)	Prostate (75)	Leukemia (brother) Breast (daughter)
	p.(Thr2607lle)						
	*Uncertain variant*						

CRC, colorectal cancer; SPS, serrated polyposis syndrome; WHO, World Health Organization.

One patient had an RNF43 c.471del G p.THR158FS variant in exon 5. There was a second‐degree relative found to carry the familial RNF43 gene variant, who had polyps but did not meet criteria for a diagnosis of SPS. There was a family history of CRC. Two unrelated patients were monoallelic MUTYH variant carriers for the pathogenic variant c1187G > A p.Gly396Asp. One of these patients also had a variant of uncertain significance in the APC gene (c.646‐4T > G). Neither patient had a personal or family history of CRC. One patient was a carrier for SMAD4 pathogenic variant c.455‐2A > G, which is associated with juvenile polyposis syndrome. However, this patient fulfilled WHO types I and II for SPS and the histology of polyps resected were not consistent with hamartomas. This patient had a personal history of left‐sided CRC aged 58. A class 4 pathogenic variant in POLD1 (c.946G > A p.Asp316Asn) was found in one patient. This patient had no personal history of CRC but had a diagnosis of endometrial cancer at the age of 54. She also had a family history of CRC and breast cancer. Two individuals had pathogenic variants in CHEK2 (c.1427C > T p.Thr476Met and c.1100delC p.Thr367fs), which is known to be associated with a predisposition to mainly breast cancers, and other cancers including CRC. Variants of unknown significance (VUS) were found in nine individuals (three MSH6, three APC, one MSH2, one BRCA2, and one NTHL1).

The genetic results led to a change in surveillance for seven patients or their families. Cascade genetic testing was recommended for at‐risk relatives of individuals with germline variants in RNF43, MUTYH, POLD1, SMAD4, and CHEK2 genes. The patient with the SMAD4 pathogenic variant was evaluated for hereditary hemorrhagic telangiectasia (HHT) and commenced upper gastrointestinal (GI) surveillance in addition to lower GI surveillance. The CHEK2 variants found also led to additional screening for breast, prostate, kidney, and thyroid cancers where appropriate.

Only 57.1% (4/7) of patients with a pathogenic germline variant and 60% (9/15) of all patients with a genetic variant in genes known to predispose to cancer fulfilled BSG criteria for genetic testing in SPS. A total of 14.28% (*n* = 1) of patients with a germline variant associated with increased CRC risk had a personal history of CRC and 57.1% (*n* = 4) had a family history of CRC.

## Discussion

Serrated polyposis syndrome is a clearly defined clinical phenotype in which only a minority of cases have been attributed to a known germline variant. In our study from a single large tertiary referral center, gene panel testing identified pathogenic germline variants in 4% of those tested. This is higher than previously reported.^33^, ^34^ The two smaller previous studies found no pathogenic variants in the specific genes tested: PTEN, SMAD4, BMPR1A, MUTYH, GREM1, and APC. In one study,^33^ the majority (83.1%) of the patients fulfilled WHO 2010 criterion 3 (equivalent to WHO 2019 criterion 2), which differs from our cohort in which 85.5% (*n* = 148) fulfilled WHO 2019 criterion 1. They found one patient to have a monoallelic MUTYH G396D variant, but they did not consider this a pathogenic variant. Although evidence is conflicting, some studies have shown individuals who are monoallelic carriers for MUTYH pathogenic variants have a small increased risk of CRC, particularly if they have a family history of CRC.^37^, ^38^, ^39^ In our study, we have considered monoallelic MUTYH pathogenic variants as an actionable variant, although the BSG does not recommend regular screening for these patients.^40^

We used gene panel‐based testing that included relevant colon cancer‐specific genes in addition to any other genes that were indicated in the patient's personal and family history. Our center currently offers a CRC gene panel consisting of 14 genes: APC, BMPR1A, MLH1, MSH2, MSH6, PMS2, MUTYH, NTHL1, POLD1 (exons 8–13), POLE (exons 9–14), PTEN, SMAD4, STK11, and GREM1 duplication. This panel has evolved over time and has changed during the 7 years since its introduction. The use of gene panel testing has resulted in a higher variant detection rate and also a widening of the clinical phenotype of known bowel cancer predisposition syndromes.^41^, ^42^, ^43^, ^44^, ^45^ It is possible that the multiple serrated polyps seen in our cohort represent an atypical phenotype of known hereditary CRC syndromes.

This study is the first to describe pathogenic CHEK2 and POLD1 variants with an SPS phenotype. CHEK2 is a moderate penetrant gene that conveys susceptibility to multiple cancers including CRC.^46^ In particular, the CHEK2 c.1100delC variant has been shown to cause an increased risk of breast cancer and the lifetime risk with this variant is approximately 25%.^47^ Women with a CHEK2 c.1100delC variant are therefore advised additional breast surveillance. CHEK2 pathogenic variants c.470T > C and c.1100delC have been associated with HNPCC and early CRC.^48^, ^49^ One patient in our cohort was positive for the pathogenic variant c.1100delC and she had a personal and family history of breast cancer, in addition to a family history of CRC in a first‐degree relative. CHEK2 was removed from the CRC/polyposis gene panel in 2016 and is no longer part of the 14‐gene panel. Polymerase proofreading‐associated polyposis (PPAP) is caused by variants in the polymerase genes POLE and POLD1 genes and is a rare cause of CRC. A pathogenic variant in the exonuclease domain of these genes causes deficient proofreading repair during replication. PPAP follows an autosomal dominant inheritance pattern and is associated with adenomatous polyposis, early CRC, and Lynch syndrome.^50^ Palles *et al*. described one patient in a family with POLD1 S478N variant with six adenomas and “multiple” hyperplastic polyps, but it is not known if they fulfilled clinical criteria for SPS.^50^ POLD1 pathogenic variants have also been found to be linked to an increased susceptibility to endometrial cancer, breast cancer, and possibly brain cancer.^51^, ^52^ The patient with a POLD1 variant in our cohort had a personal history of endometrial cancer in addition to a family history of CRC and breast cancer. It has been recommended that patients with POLD1 and POLE variants undergo regular colonoscopic and gastroscopic surveillance, in addition to screening for endometrial cancer.^53^

One patient in our study had a previously unreported pathogenic variant in RNF43 (c.471delG p.Tyr158fs). This deletion is predicted to cause a frameshift in exon 5 leading to premature termination in translation. Other premature termination translation variants associated with serrated polyposis and CRC have previously been reported.^19^, ^20^, ^21^ RNF43 is not included in the polyposis gene panel in Oxford, but sequencing was performed due to a family history of serrated polyposis and an RNF43 variant. Of note, neither RNF43 nor CHEK2 is included in the current UK Cancer Genetics Group panel testing for polyposis patients.

There has been some concern that an increase in genetic testing with gene panels may lead to high levels of anxiety among patients. Reassuringly, any short‐term anxiety or distress experienced on receiving positive genetic test results does not appear to last in the medium term to long term.^54^, ^55^ Careful pre‐test counseling is important for managing expectations and reducing possible distress.^56^ Carriers of variants in moderate penetrant genes, such as the CHEK2 gene, have been shown to experience increased distress, likely due to the lack of clarity on cancer risk and optimal surveillance.^57^ It is important that variants of uncertain significance are appropriately interpreted and do not result in unnecessary changes in management. Perhaps surprisingly, patients with these variants do not appear to have increased levels of uncertainty.^57^, ^58^

There are some limitations to this study. Firstly, only 42.2% of patients underwent genetic testing. The decision for referral to clinical genetics was based on the patient's personal and family history, in addition to patient preference. In this way, we may have underestimated the yield of pathogenic variants in genes that are known to predispose to CRC. Secondly, a uniform gene panel was not used for all patients. It is possible that patients who had negative genetic testing for targeted genes or smaller gene panels would be positive for a pathogenic variant using the current 14‐gene panel.

Colorectal cancer panels are limited and hamstrung by a lack of understanding of genetic drivers in SPS. The germline variants that predispose to this common pathological and potentially mixed phenotype are not well known; thus, there is need for a comprehensive whole genome study approach to update old CRC panel testing and to better link Mendelian genotype with phenotype. Also, for the majority where no germline variant is found, the presumption is that this is predominantly a polygenic condition.

In conclusion, 9.6% (7/73) of our tested SPS cohort had a pathogenic variant in a gene known to predispose to CRC and this led to a change in management for the patient or their family in all cases. Just over half of these patients who were carriers of pathogenic variants fulfilled BSG criteria for genetic testing. The rationale behind this BSG guidance is to rule out other intestinal polyposis syndromes, which may present with multiple serrated polyps. We propose that the SPS phenotype is under‐recognized as a clinical presentation of hereditary colorectal syndromes and that all patients fulfilling the WHO criteria for SPS be seen in a family cancer clinic to discuss surveillance strategy and CRC risk, and to consider referral to Clinical Genetics for genetic counseling and gene panel testing.
